# Kidney Perfusion as an Organ Quality Assessment Tool—Are We Counting Our Chickens Before They Have Hatched?

**DOI:** 10.3390/jcm9030879

**Published:** 2020-03-23

**Authors:** Julie De Beule, Ina Jochmans

**Affiliations:** 1 Laboratory of Abdominal Transplantation, Transplantation Research Group, Department of Microbiology, Immunology, and Transplantation, KU Leuven, 3000 Leuven, Belgium; julie.debeule@kuleuven.be; 2 Department of Abdominal Transplant Surgery, University Hospitals Leuven, 3000 Leuven, Belgium

**Keywords:** machine perfusion, kidney preservation, quality assessment, viability assessment, vascular resistance, injury biomarkers, kidney perfusion

## Abstract

The final decision to accept an organ for transplantation remains a subjective one. With “poor organ quality” commonly cited as a major reason for kidney discard, accurate, objective, and reliable quality assessment is essential. In an era of increasingly higher-risk deceased donor kidneys, the catch is to accept those where the risk–benefit scale will tip in the right direction. Currently available assessment tools, such as risk-scores predicting outcome and zero-time biopsy, perform unsatisfactory, and assessment options during static cold storage are limited. Kidney perfusion technologies are finding their way into clinical practice, and they bring a new opportunity to assess kidney graft viability and quality, both in hypothermic and normothermic conditions. We give an overview of the current understanding of kidney viability assessment during ex situ kidney perfusion. A pragmatic framework to approach viability assessment is proposed as an interplay of three different compartments: the nephron, the vascular compartment, and the immune compartment. Although many interesting ways to assess kidney injury and function during perfusion have been proposed, none have reached the stage where they can reliably predict posttransplant outcome. Larger well-designed studies and validation cohorts are needed to provide better guidance.

## 1. Introduction

Data from Eurotransplant, the United Kingdom, and the United States of America show that, every year, about 15% of kidney transplant candidates die on the waiting list while 15 to 20% of deceased donor kidneys offered are not transplanted [1,2,3,4,5]. Clearly, some kidneys are rightfully declined because of contraindications for transplantation such as cancer, anatomical defects, or infectious diseases [1,2]. However, a substantial number would provide a favourable risk–benefit ratio to some waitlisted patients [1]. The catch is to select those cases where the risk–benefit scale will tip in the right direction. 

With “poor organ quality” commonly cited as a major reason for kidney discard [2,5,6], it is clear that accurate, objective, and reliable quality assessment is essential. This is especially important in an era with increasingly higher-risk deceased donor kidneys of advanced age and increasing comorbidities that are more susceptible to ischemia reperfusion injury. Severe ischemia reperfusion injury is associated with primary non-function (PNF) and cortical necrosis on biopsy [7,8,9]. In other cases, ischemia reperfusion injury might take a milder course with kidney function recovering over time. However, mild to moderate injury is not as innocuous as it may sound. Delayed graft function (DGF), arbitrarily defined as the need for dialysis in the first week posttransplantation, is associated with the ischemic hit the kidney undergoes [9,10,11] and also with a significantly higher risk of acute rejection and graft loss [12]. Predicting the occurrence of such events prior to transplantation could avoid futile transplants or allow the team to make a more informed decision when a higher-risk kidney offer comes.

Studies suggest that the final decision taken by the transplant team to accept an organ for transplantation remains a subjective one [5,13,14]. With most kidneys preserved by static cold storage, the options for viability and quality assessment of the graft are limited. This evaluation process at time of offer relies on pre-donation review of donor characteristics, donor circumstances and biochemistry, visual appraisal of the organ, and—depending on local practice—results of a zero-time kidney biopsy. Zero-time biopsies are one of the primary reasons of discard in centres relying on these histological findings, while there is a lack of validated scoring systems in combination with poor predictive power [1,15,16]. Considerable effort has gone into the development of risk stratification scores that predict the occurrence of DGF [17,18,19] or graft survival after kidney transplantation [20,21,22,23]. None of the existing models provide adequate predictive power and should therefore be seen as tools that provide additional information that might guide but should not dictate the decision to transplant [23,24]. There is also some suggestion that there might be an unexpected, harmful “labeling effect” of reporting risk stratification scores [25,26]. Although the zero-time kidney biopsy aims to reduce uncertainty, decisions solely based on histology should be avoided [16,27].

**Figure 1:**
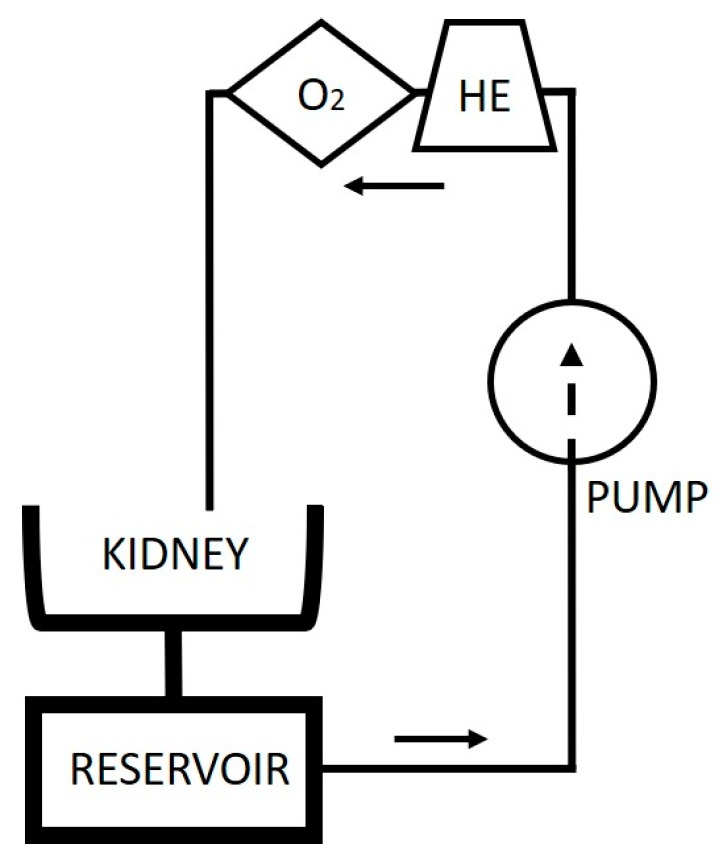
A schematic of a kidney perfusion circuit: The presence of a heat exchanger (HE) and gas exchanger (O_2_) depends on the perfusion mode. HE, heat exchanger; O_2_, gas exchanger.

A new opportunity to assess kidney graft viability and quality presents itself now that kidney perfusion technologies are finding their way into clinical practice [28]. These technologies (often referred to as machine perfusion) provide continuous circulation of a perfusion solution through the kidney vasculature (Figure 1). This perfusate can be heated or cooled, allowing normothermic, subnormothermic, or hypothermic perfusion. Inclusion of a gas exchanger provides the opportunity to oxygenate the perfusion solution or to give any other gaseous treatment to the organ. “Dynamic” in contrast to “static” organ preservation allows us to study different aspects of the organ during perfusion as the kidney metabolism can be maintained [28].

In this review, we give an overview of the current knowledge on kidney viability assessment during kidney perfusion strategies. We focus on clinical data or data generated in large animal studies or studies making use of kidneys that were not suitable for transplantation.

## 2. Kidney Perfusion Strategies: The Forest Through the Trees

With increasing use of higher-risk organs, interest in dynamic preservation methods is revived. A multitude of dynamic preservation strategies are the subject of current research and some have already been implemented in routine clinical practice. Mostly, dynamic preservation methods are categorized by their target temperatures as this directly reflects how organ metabolism is maintained. The facilitation of organ assessment is also closely linked with the temperature at which organs are perfused, as cellular metabolism is directly correlated with temperature [29].

### 2.1. Hypothermic Kidney Perfusion

Hypothermic dynamic preservation slows down cellular metabolism and counteracts undesirable and detrimental effects of ischemia. It combines low temperature (4–10 °C) with an acellular colloid-containing preservation solution that is pumped through the kidney vasculature [28]. Whether hypothermic perfusion results in improved posttransplant outcomes compared to static cold storage has been the subject of a number of randomised controlled trials (RCT), systematic reviews, and meta-analyses [30,31,32,33,34,35]. The most recent of these was a Cochrane systematic review [33]. The meta-analysis showed that hypothermic kidney perfusion reduced DGF rates for both kidneys donated after brain death (DBD) and those donated after circulatory death (DCD), even though there remains some controversy with regard to the effect of hypothermic perfusion in DCD kidneys [33,34,36]. Fourteen of the 16 studies, with a total of 2266 patients, focused on DGF as primary outcome. In this Cochrane review, evidence of two RCTs that evaluated the effect on graft loss was considered strong enough to assume a positive effect of hypothermic perfusion on kidney graft survival [31,33,35]. The largest RCT, performed by Moers et al., contributed the most in both analyses and represents generalizable results from DBD (standard criteria donors (SCD) and extended criteria donors (ECD)) as well as DCD kidneys from 60 different centres throughout Europe [31]. As some evidence shows targeting low pressures could avoid pressure-related injury, pulsatile renal artery perfusion at controlled pressures of 25–30 mmHg is regarded best for hypothermic kidney perfusion [30,31,34,37,38,39].

Because of the low temperatures and severely reduced metabolic rate, hypothermic perfusion is likely not the ideal setting to evaluate residual organ function [28]. However, assessment of the severity of damage the organ has sustained in the course of the donation process might be possible. Indeed, brain death is associated with activation of the endothelium and innate immune system and leads to tubular injury [40,41,42]. In DCD donation, the kidney has already been exposed to an agonal phase of low perfusion pressure and hypoxia with additional warm ischemia at the time of circulatory arrest [43]. 

Although hypothermia slows down metabolism, there is no complete metabolic standstill. Preclinical studies showed that cellular respiration continues during hypothermic perfusion, resulting in oxidative stress [44,45]. Active oxygenation during hypothermic perfusion might therefore have a positive effect on kidney graft function, and this is the subject of two RCTs initiated by the Consortium on Organ Preservation in Europe (COPE) [28]. In this setting, where oxygenation supports a limited metabolism, it might be that assessment of organ function in hypothermic conditions becomes possible.

### 2.2. Normothermic Kidney Perfusion

The aim of normothermic dynamic perfusion (35–37 °C) is to restore normal cellular processes. Normothermic preservation mandates an oxygenated perfusate and the use of an oxygen carrier. Although this oxygen carrier is usually red blood cells, acellular perfusates are under investigation, making use of hemoglobin-based oxygen carriers [46]. At normothermia and in the presence of oxygen, cellular metabolism resumes, and therefore it is likely that assessing both organ injury as well as residual function can take place during normothermic organ perfusion [28].

At current, most of the available evidence investigates normothermic kidney perfusion during a brief period (usually 1 hour) before transplantation. In a pilot study of ECD kidneys, the Nicholson group retrospectively compared the effect of 1-hour preimplantation normothermic perfusion with static cold storage [47]. They found a significantly lower DGF rate in the 18 kidneys that had undergone a brief period of normothermic perfusion and found no effects on graft and patient survival. Subsequently, an RCT comparing 1-hour preimplantation normothermic perfusion with static cold storage of DCD kidneys was started [48]. This study was identified as the only ongoing trial about normothermic kidney perfusion by the Cochrane systematic review [33,48]. The same group showed that kidney assessment during warm perfusion might lead to transplantation of initially discarded DCD kidneys [49]. Extending normothermic perfusion to longer periods (2 to 8 hours) has been explored in pig studies [50,51,52,53,54]. Kidneys not suitable for transplantation have been normothermically perfused for 24 hours, making use of urine recirculation, though not transplanted [55]. Prolonged normothermic perfusion might offer improved preservation and assessment as well as time to repair organ injury [56].

The composition of the perfusate is likely very important, and a variety of protocols are currently used. Although an in-depth analysis of the different perfusates is outside the scope of this review, it is worthwhile to note that most perfusates for normothermic perfusion use red blood cells in combination with colloids and crystalloids, a vasodilator, nutrients, and usually also antibiotics. 

### 2.3. Subnormothermic Kidney Perfusion

Subnormothermic dynamic preservation (20–25 °C) aims to avoid cold-induced injury without increasing metabolism to a level at which intense oxygenation requires an oxygen carrier [28]. Indeed, both acellular and cellular perfusates have been used, showing that subnormothermic perfusion without an oxygen carrier is feasible [57,58]. Until now, subnormothermic kidney preservation has only been tested in DCD models of ex situ perfusion of pig kidneys [57,58,59,60]. Hoyer et al. found improved kidney function after subnormothermic compared to oxygenated hypothermic kidney perfusion [60].

As oxygen is provided and temperature is increased, subnormothermic perfusion might be useful for organ assessment, though no studies looking into this are available.

### 2.4. Controlled Oxygenated Rewarming

During controlled oxygenated rewarming (COR) a statically cold-stored organ is slowly rewarmed until 20 °C to avoid a sudden heat shock, which occurs when an ice-cold organ is suddenly rewarmed to body temperature at time of reperfusion in the recipient [28]. In a pig autotransplant model, COR of DCD kidneys resulted in improved posttransplant kidney function when compared to static cold storage [61]. Another recent preclinical trial using porcine DCD kidneys suggests COR improves mitochondrial recovery [62]. A report on one ECD kidney being progressively rewarmed and successfully transplanted has been published [63]. 

### 2.5. Combining Perfusion Conditions

All the conditions described above can be potentially combined. For example, the combination of hypothermic perfusion for preservation purposes and normothermic perfusion for assessment purposes has been reported by Kabagambe et al. [64]. In this study, seven kidneys that had been initially statically cold-stored, placed on hypothermic perfusion at the recipient centre, but eventually found not suitable for transplantation were placed on normothermic perfusion but not transplanted. Similar approaches have been piloted in liver, resulting in successful transplants after viability assessment, suggesting that this approach also lends itself to organ assessment [65].

## 3. Viability Assessment of the Kidney: Compartmentalize the Complex Kidney Structure

The kidney is a complex puzzle of different cell types carrying out a multitude of life-sustaining biochemical functions that are, in broad terms, the filtration and cleansing of blood and the production of the hormones renin and erythropoietin. If we were to simplify this complex puzzle, we could pragmatically differentiate different kidney compartments: (1) the nephron as the functional unit of the kidney; (2) the vascular compartment of the kidney with its unique capillary beds: the peritubular capillaries and the vasa recta [66]; and (3) the immune compartment with its resident immune cells. Although conceptualising the kidney as such allows us to consider and describe how viability and quality of each of these compartments might be assessed, it is an oversimplification of the multifaceted interactions of these compartments and the way they are intertwined. For example, the nephron, which is composed of the glomerulus and the cortical and medullary tubular systems, can be regarded as an appendage of the renal blood vascular system, attached to the blood supply for servicing purposes [67].

Below, we provide a short overview on some essentials relating to the kidney’s morphology and function. We also summarize the current knowledge on suggested viability markers measured during kidney perfusion and approach this by using the conceptual framework of the three kidney compartments.

### 3.1. The Nephron

In the clinical setting of transplantation, the interplay between the severity of kidney injury of different cell types, residual kidney function, as well as the kidney’s regenerative response—or lack thereof—is crucial. Indeed, some of the kidney’s cell types, like the podocytes of the glomerulus, are terminally differentiated cells that fail to divide, and when injured, these cells are not replaced [68]. Others, like the proximal tubular cells, have significant regenerating capacity, though, when injury is severe, repair can be incomplete leading to chronic injury [69,70]. Ideally, viability markers would not only assess severity of injury but also should focus on whether the kidney will function or regain function posttransplantation.

#### 3.1.1. Nephron Function

In vivo, the glomerulus is responsible for ultrafiltration and regulation of renal blood flow. The tubuli combine their reabsorbing capacity for ions and nutrients with important secretory and excretory functions to change the composition of the ultrafiltrate with final modifications to the urine in the collecting ducts. Whether and how in vivo findings translate to the setting of kidney perfusion is unclear. When considering how nephron function might be determined during kidney perfusion, it is important to consider the physiological principles underlying the glomerular and tubular function. The glomerular filter of an intact nephron consists of a tangle of blood vessels between the afferent and efferent arterioles—the glomerular tuft—that possess a fenestrated endothelium. Together with the glomerular basement membrane and a slit diaphragm, with pores formed by interdigitating feet of podocytes, the fenestrated endothelium forms a unique barrier with a specific permselectivity [66,71]. Molecules with low molecular weight (<5500 Da) and small effective molecular radius are easily filtered and tend to appear in the filtrate in the same concentration as in the plasma [72]. As the glomerular filter is negatively charged, the electrical charge of a molecule also influences whether it can pass the filter. Certain large molecules, including some proteins, may pass through the filter and into the urinary space, where they require reabsorption by the tubular system [67]. In vivo, it is generally accepted that albuminuria or proteinuria can be interpreted as sign of a damaged glomerular filter [73]. 

In addition to permselectivity of the glomerular barrier, interplay between both hydrostatic and oncotic pressures further determines glomerular filtration rate (GFR) and substance concentrations in the initial filtrate. In vivo, hydrostatic pressure is determined by the arterial blood pressure in the glomerular capillaries and is regulated by the contraction status of the afferent and efferent arterioles. Associated oncotic pressures in these capillaries are maintained mainly by the intravascular albumin concentration, which is on its turn again influenced by renal blood flow. If renal blood flow drops, oncotic pressures will rise in the glomerular capillary.

In vivo, nephron function is mostly measured by estimating the GFR, often by determining a creatinine clearance. A GFR is only a meaningful reflection of nephron function in steady state [74,75,76]. The fact that creatinine is also partially secreted by the tubuli should not be overlooked [74]. Creatinine can easily be added to the closed circuit of normothermic perfusion, either as a bolus at the start or intermittent spikes or as continuous infusion, if so desired. Monitoring of creatinine concentrations in the perfusate and urine allow the calculation of a creatinine clearance. Absolute creatinine clearance values during normothermic perfusion are usually very low [77]. Whether changes in perfusate creatinine concentration during perfusion are predictive of posttransplant function is unknown. Furthermore, although the physical properties of the filter remain the same when a healthy kidney is perfused ex situ, the perfusate composition and perfusion pressures (pump pressures) will change hydrostatic and oncotic pressures and therefore influence filtration and ultimately “urine” production during kidney perfusion. Indeed, if perfusion pressures are maintained high enough with low oncotic pressures, theoretically every kidney can be forced to produce a filtrate, which does not necessarily mean that the filter function is intact. Furthermore, the glomerular tangles of blood vessels need to be open and the impact on the filtration barrier of ischemia reperfusion injury at cellular level is not clear. Mild ischemia is associated with damage to the charge selectivity of the glomerular filtration barrier [78]. If ischemic periods are longer, damage to the filter becomes nonselective [73,79]. 

In vivo, high fractional sodium excretion (FeNa) (usually >1%) is suggestive of tubular necrosis [80]. Several normothermic perfusion models, making use of pig kidneys or kidneys found not suitable for transplantation, report FeNa with a high variability and a wide range (5%–200%) [81,82,83,84,85]. Extrapolating in vivo findings, it seems logical that the lower the FeNa during normothermic perfusion, the better tubular function is preserved. However, no information on how to interpret exact FeNa values during kidney perfusion is available and whether they are predictive of posttransplant outcome is unknown. We do know that a linear relationship exists between sodium reabsorption and oxygen consumption because sodium reabsorption is an energy-dependent and therefore oxygen-dependent process [86]. When oxygen is administered during hypothermic perfusion, oxygen consumption of kidneys showed a high correlation with post reperfusion GFR measured in a Langendorff circuit [87]. Therefore, measuring oxygen consumption could be a potential viability marker. Indeed, oxygen consumption is measured in different models as a marker of kidney metabolic activity [81,84,85,88]. What limits direct comparison between studies is the use of a multitude of formulas to calculate oxygen consumption. Existing formulas require correction of hemoglobin concentration, and it is unclear how this needs to change when kidneys are perfused with other oxygen carriers or an acellular perfusate. It is also important to correct for temperatures other than body temperature. Furthermore, there is evidence that oxygen consumption in the kidney during normothermic perfusion is dependent on the oxygen concentrations offered to the kidney [81]. Metabolomics analysis could offer additional information. When Patel et al. administered fully labelled ^13^C-glucose to the circuit of hypothermically perfused pig kidneys with or without oxygenation, they could show higher newly synthesized glutamate concentrations when kidneys received oxygen, indicative of a more active aerobic metabolism [89].

#### 3.1.2. Nephron Injury Markers

Traditionally, cellular injury is assessed on histology. However, there is a delay between cellular injury and histological changes, and in hypoxic acute kidney injury, there is a relative uniformity of opinion that overt tissue injury is limited [90,91]. When tubular necrosis, as defined by cell loss is present, it seems to affect distal tubular medullary segments (medullary thick ascending limbs and medullary collecting ducts) to a larger extent than proximal tubular segments located in the outer medulla or the cortex [92]. Furthermore, injured and dying cells shed or leak cytosolic and mitochondrial content that could be used as injury markers in urine or perfusate [65,93]. Additionally, there seems to be an inherent sensitivity of proximal tubular cells to warm ischemic injury whereas cold ischemia elicits distal tubular injury, with different patterns of response [90]. While continuous supply of oxygen is obligatory for proximal tubular cell viability, the cells of the thick ascending limb of the distal tubuli are quite resistant to hypoxia, as long as energy expenditure for transport activity is withheld [94]. However, transport activity is the major factor governing thick ascending limb injury during oxygen deprivation [95], probably due to high mitochondrial respiration, leading to a rapid decline of energy stores and the formation of reactive oxygen species [90]. Thus, with the complete cessation of GFR and tubular transport, induced by warm ischemia, the cells of the thick ascending limbs maintain viability while proximal tubular cells are quickly affected [90]. Cold preservation encountered in experimental transplantation leads to a better preservation of proximal tubular cells and a more pronounced damage to the thick ascending limb upon reperfusion [90]. As different markers are released from different cells types (e.g. kidney ischemia molecule-1 (KIM-1) originates from proximal tubular cells and neutrophil gelatinase-associated lipocalin (NGAL) originates from the thick ascending limb), biomarker patterns might give insight into injury patterns.

##### Hypothermic Perfusate Injury Markers

Individual studies and a recent systematic review concluded that existing studies looking at the use of perfusate biomarkers during hypothermic kidney perfusion are limited in quality and have not yielded biomarkers that are able to predict posttransplant outcomes with sufficient accuracy to support routine clinical use [96,97,98,99]. Glutathione S-transferase (GST), as total-GST (t-GST) or its isoforms (alpha-GST and pi-GST), was the most commonly evaluated biomarker, followed by lactate dehydrogenase and lactate levels [97,98,100,101,102,103,104,105,106]. At least two different articles reported on one or more of the following markers: alanine-aminopeptidase [98,107], fatty acid binding protein (FABP, in its isoforms heart-FABP and liver-FABP) [97,98,99,107], NGAL [97,99,108], interleukin-18 [97,99], lipid peroxidation products [104,109], redox-active iron [97,103], and perfusate ionized calcium [110,111]. Single-study results were present for histones H3 [112], KIM-1 [99], aspartate transaminase (AST) [98], matrix metalloproteinase 9 and 2 [108], n-acetylglucosamine [98], micro-RNA 21 [113], and perfusate proton nuclear magnetic resonance spectroscopy [114]. Nothing definite could be concluded with regard to the prediction of PNF rates because of low occurrence rates and small sample sizes [96]. Nevertheless, in a cohort of 335 hypothermically perfused DCD kidneys, Hoogland et al. showed that lactate dehydrogenase and interleukin-18 were independently associated with PNF [97]. Diagnostic accuracy for PNF, however, was poor [97]. Although GST is, at current, the most promising biomarker for predicting DGF during hypothermic perfusion [96], its predictive ability is moderate at best [96,97,98,99]. Parikh et al. reported biomarkers in the largest prospective cohort of 671 DBD and DCD kidneys [99]. NGAL, liver-FABP, and interleukin-18 values were significantly associated with DGF [99]. Most values for KIM-1 were below detection limits [99]. NGAL and H-FABP were also independently associated with lower estimated GFR at 6 months, but predictive accuracy was moderate at best [99].

Flavin mononucleotide (FMN) has been proposed as a promising predictive marker for early allograft dysfunction and early graft loss when measured during hypothermic oxygenated liver perfusion [115]. FMN has also been reported as a marker of mitochondrial function in a pig autotransplant model where kidneys were subjected to 30 minutes of warm ischemia and 22 hours of cold perfusion with varying oxygenation [93]. FMN is non-covalently bound to a subunit of the mitochondrial complex I and therefore an interesting marker [116]. Dissociation of reduced FMN is seen during ischemia when reverse electron transferring takes place and mitochondria oxidize succinate [116]. In membranes of isolated mitochondria derived from brain tissue 35 minutes after ischemia reperfusion injury, a significant drop in non-covalently bound FMN was identified [117]. Additionally, free FMN reacts non-enzymatically with oxygen and easily produces reactive oxygen species that are harmful to cells and tissue [116]. FMN can also be found in nitric oxide synthase, though perhaps in lower concentrations than expected in mitochondria [118]. Whether perfusate FMN reflects mitochondrial function or injury and whether it can predict outcome after kidney transplantation is unknown.

Lastly, perfusate cell profiles might prove an interesting avenue for future research as it was recently shown that parenchymal cells are released from organs under non-proliferative pathological conditions, correlating with the degree of ischemic injury [119]. Indeed, in hypothermic rat liver perfusion, it has been shown that hepatocytes, sinusoidal endothelial, stellate, and liver-specific immune cells are released into perfusates with different cell profiles between those released from healthy livers compared to those of cold ischemic ones [119].

##### Normothermic Perfusate Injury Markers

Few data are available regarding perfusate or urine biomarkers during normothermic kidney perfusion, and none have been investigated or validated in large cohorts of kidney transplants. In a series of 56 discarded human kidneys that underwent 1 hour of normothermic perfusion at the end of cold storage, NGAL and endotheline-1 were associated with higher kidney quality scores but the kidneys were not transplanted [84]. This kidney quality score was developed by the Nicholson group and combines macroscopic appearance, renal blood flow, and urine output during 1 hour of normothermic perfusion [120,121]. The kidney quality score is detailed below (Table 1). Biomarkers determined in the perfusate of pig kidneys undergoing 8 hours of normothermic perfusion after exposure to 0, 30, or 60 minutes of warm ischemia showed that markers of acid-base homeostasis (pH, HCO3–, base excess) correlated with posttransplant renal function [50]. Similarly, lactate and AST were lowest in non-injured grafts and levels correlated with posttransplantat kidney function [50].

### 3.2. The Vascular Compartment

As described above, the renal vascular system has a highly specialized preliminary capillary network, the glomerular tuft, which receives blood from an afferent arteriole and which is the site of filtration of waste products from plasma [67]. The kidney also has a second capillary system arising from the efferent arteriole, which varies in structure and function according to its location within the kidney [67]. On the one hand, the efferent arteriole divides into a complex capillary system which runs in the interstitial spaces between the components of the system of cortical tubules [67]. These peritubular capillaries are in intimate contact with the tubules and are therefore ideally placed to take up any substances reabsorbed from the glomerular filtrate by tubular epithelial cells [67]. On the other hand, the capillary system originating from efferent arterioles leaving the juxtamedullary glomeruli in the deep cortex divide into the vasa recta [67]. The vasa recta are long, thin-walled vessels which run straight down into the medulla alongside the medullary components of the tubular system [67]. The vasa recta play an important role in the ionic and fluid exchanges that take place in the medulla [67].

#### 3.2.1. Endothelial Dysfunction

In vivo, the basal functions of the vascular endothelial cells maintain blood fluidity, regulate blood flow, control vessel wall permeability, and quiesce circulating leukocytes [122]. During inflammation the endothelial cells fail to adequately perform some or all basal functions which is referred to as “endothelial cell dysfunction” [122]. In the setting of transplantation, ischemia reperfusion leads to a sterile inflammation in which infiltrating leukocytes and vascular endothelial cells have a major role, changing their phenotypes to support various phases of the inflammatory process [122]. Indeed, microvascular endothelial cells at a site of inflammation are both active participants in and regulators of inflammatory processes [122]. An excellent review by Pober et al. summarizes the response of the microvasculature to inflammation, which consists of type I (rapid, over a period of hours) or type II activation (slower response, depending on new gene expression) responses of endothelial cells [122]. The type I response is mediated by activation by ligands of heterotrimeric G-protein coupled receptors such as histamine, and type II activation is mediated by inflammatory cytokines, such as Tumour Necrosis Factor α (TNFα) or Interleukin-1. Time-dependent changes in molecules expressed by type-II-activated endothelial cells underlie the change in inflammation from neutrophil-dominated to mononuclear-cell dominated responses [122]. Adaptive immunity, through cytokines such as Interferon γ (IFNγ) or Interleukin-4, may superimpose changes on type-II-activated endothelial cells that enhance Th1-type or Th2-type polarized inflammatory reactions [122].

How these processes change in the setting of kidney perfusion, where circulating leukocytes are usually removed from the perfusate, is not known, and it would be worthwhile to investigate how the endothelium responds to different kidney perfusion strategies. Indeed, endothelial cell injury and death, with disruption of the endothelial cell lining, favour thrombosis, which in combination with the no-reflow phenomenon, cause an increase in vascular resistance that reflects endothelial damage and viability [123,124]. Local disturbances in blood flow reduce endothelial cell nitric oxide (NO) production and may thus contribute to vasoconstriction as well as to local leukocyte or platelet activation [122]. Tietjen et al. showed the presence of red blood cell plugs in both cortex and medulla during 4 hours of normothermic perfusion of discarded human kidneys, and these likely contribute to injury and the no-reflow phenomenon [125]. Oedema, in part, the result of leaky endothelium, may also influence blood flow to an organ [124]. To counteract vasoconstriction and the no-reflow phenomena, the need to add vasodilatory agents to the perfusion circuit has been recognized. Different vasodilators or combinations of them (verapamil, epoprostenol, glyceril trinitrate, endothelin-1 antagonist, etc.) with various acting mechanisms have been added to organ perfusion circuits [50,55]. Whether one of these is superior in the setting of kidney perfusion is unknown; however, in normothermic liver perfusion, endotheline-1 antagonist or verapamil resulted in higher hepatic flows and lower AST levels [126]. In kidneys, different vasodilatory agents have only been tested during hypothermic perfusion. Adding prostaglandin E1 to the perfusate was found to result in increased renal flow and decreased renal resistance during hypothermic perfusion. Moreover, kidneys treated with prostaglandin E1 was associated with improved early graft function when compared to adding verapamil, trifluoperazine, or mannitol [127].

Bath et al. recently demonstrated the importance of evaluating responsiveness of endothelial cells to acetylcholine [82]. As healthy endothelial cells respond to vasoactive substances, the disappearance of such a response gives information about endothelial dysfunction. In a porcine model of normothermic kidney perfusion, kidneys that had been exposed to 2 hours of warm ischemia did not show any vasodilating capacity when exposed to acetylcholine, suggesting irreversible injury of endothelial cells. On the other hand, kidneys exposed to 16 hours of cold ischemia only showed a diminished response to acetylcholine [82].

#### 3.2.2. Vascular Resistance During Perfusion as a Viability Marker

In vivo, ischemia reperfusion injury leads to the activation of inflammatory pathways, to production of radical oxygen species, and to activation of vasoactive compounds that results in endothelial cell damage and increased vascular resistance [7]. After reperfusion, tubular congestion and oedema further contribute to reduced renal blood flows [7]. Both flow (when there is a constant perfusion pressure) and renal vascular resistance evaluated during kidney perfusion could therefore be an interesting viability parameter. In this light, it is important to consider that kidney perfusion is mostly pressure-controlled with arterial pressures maintained at reference values. For hypothermic perfusion, these are 25–30 mmHg; for normothermic perfusion, they currently vary from protocol to protocol between 60–110 mmHg. Depending on intravascular resistance, which tends to be raised in damaged organs, renal blood flow will change with changing perfusion pressures. Therefore, both flow and pressure should be reported simultaneously and preferentially resistances should also be given. Furthermore, as the renal vasculature might respond differently to different types of vasodilator, as described above, it seems that the type of vasodilator used in a particular normothermic setups might play a role in absolute values of flow and resistances. This should also be kept in mind when comparing findings from experimental and clinical studies.

Although vascular resistance measured during clinical hypothermic perfusion has been shown to be an independent risk factor for DGF, PNF, and graft survival, it is discouraged to use resistance measures as the sole basis for organ acceptance decision making [24,99,128,129]. In a cohort of 440 DCD kidneys, de Vries et al. could show that renal vascular resistance at the beginning of perfusion was an independent risk factor for PNF [128]. However, predicative accuracy was modest [128]. In a cohort of 336 kidneys (both DBD and DCD), we showed that renal vascular resistance at the end of hypothermic perfusion was an independent predictor of DGF and graft survival, though predictive accuracy was also modest at best [129]. In the largest study evaluating the value of vascular renal resistance, Parikh et al. confirmed these findings. They also found that renal vascular resistance is an independent predictor of estimated GFR at 6 months with equally modest predictive accuracy [99]. Despite these studies, associations between higher discard rates and the use of pump parameters as discard criteria are reported [130].

Little is currently known about the meaning of flow and resistance during normothermic perfusion of the kidney, although with increasing perfusion time, flow is usually seen to increase while resistance drops if perfusion pressures are maintained constant. Flow—not resistance—is one of the parameters of the kidney quality assessment score developed by the Nicholson group [123]. This score combines macroscopic appearance, renal blood flow, and urine output during 1 hour of normothermic perfusion that is performed at the end of cold storage [120,121]. The score was developed in a series of kidneys unsuitable for transplantation. Receiver-operating characteristic curves were used to determine thresholds of renal blood flow and urine output to differentiate between macroscopic grades I (well-perfused appearance) and II (patchy appearance during perfusion) versus grade III (global mottled appearance during perfusion). This led to cut-off values for renal blood flow (50 ml/min/100 g) and urine output (43 ml) (Table 1) [121]. In later work, five out of eight initially discarded human kidneys that scored a kidney quality assessment score between 1 and 3 were transplanted successfully [49]. Although it is recognized that this score needs further development and validation in large cohorts, it is proof that normothermic perfusion has the potential to increase the number of kidney transplants by providing additional information and perhaps some assurance to the transplant teams [49]. It is also important to note, with previous remarks in mind, that absolute values of renal blood flow and urine production will depend on perfusion pressure settings and perfusate composition (additives and oncotic pressures), and therefore, scores such as the kidney quality assessment score might not necessarily be transferrable to other settings where different pumps, perfusate compositions, and additives are used.

### 3.3. The immune cell compartment

Ischemia reperfusion injury causes a sterile inflammation that triggers activation of the innate and adaptive immune system and recruitment of leukocytes [131]. Additionally, the endothelium and epithelial cells play a key role [122]. Furthermore, cytokines and chemokines initiate and re-enforce the primary leukocyte response and elicit a detrimental vicious cycle [132]. These signalling molecules are produced by leukocytes and endothelial cells. Injury to tubular epithelial cells has also been shown to influence upregulation of cytokines and chemokines by means of three key cellular pathways [133]. Damage associated patterns (DAMP’s), like high-mobility group box-1 (HMGB-1), are endogenous ligands binding to Toll like receptors (TLR), resulting in activated cellular pathways with production of proinflammatory cytokines and chemokines [131,132]. TLR-activated pathways also interact with the complement system [133]. Additionally, sphingosine-1-phosphate, produced by endothelial cells, platelets, and leukocytes, binds to G-protein-coupled receptors on tubular epithelial cells which can affect mitochondrial structure and function and can mediate proapoptotic and proinflammatory effects [133]. Finally, hypoxia inducible factors are known to induce glycolytic metabolism and to regulate production of vascular endothelial growth factor (VEGF) and erythropoietin (EPO) [131,133].

These are all events that take place in vivo, and whether or how they are changed during ex situ kidney perfusion is not known. Indeed, in most cases, ex situ perfusion takes place with a perfusate that is either acellular or red blood cell based. Removal of circulating leukocytes from the perfusate is thought to minimize inflammation during normothermic perfusion compared to perfusion with whole blood. However, it is interesting to note that a perfusate based on whole blood showed a lower AST release compared to a red-cell-only-based perfusate in a model of pig normothermic liver perfusion [134]. This indeed illustrates that the ischemia reperfusion cascade, its feedback loops, and its effects during ex situ perfusion are incompletely understood.

Furthermore, despite the absence of circulating leukocytes during perfusion with an acellular or red-cell-only-based perfusate, resident leukocytes are released during kidney perfusion. These resident leukocytes are abundantly present in the kidney as was shown when the immune compartment of foetal and mature kidneys was mapped using single cell RNA sequencing technology [135]. Mononuclear phagocytes, neutrophils, mast and dendritic cells, CD4- and CD8-positive T cells, and Natural Killer (NK) cells were present [135]. The cortex or corticomedullary area harbours almost all B cells, whereas mononuclear phagocytes were mainly identified in the medullary regions during depth analyses [135]. When adding a leukocyte filter to the circuit of normothermic kidney perfusion with a red-cell-based perfusate, CD3+- T cells (40%), neutrophils (17%), and B cells (5%) were caught by the filter [136]. Furthermore, flow cytometry analyses of perfusate samples taken 2–3 minutes after start of normothermic kidney perfusion confirmed a significant efflux of leukocytes (CD45+) [83]. Granulocytes represented the most abundant group, next to CD14+ monocytes, CD3+ T cells, CD19+ B cells, and CD56+ NK cells [83]. No dendritic cells could be identified [83]. It is unclear what the implications of the presence and release of resident leukocytes are and whether they might be predictive of posttransplant outcomes. However, the use of a leukocyte filter during 3 hours of ex situ normothermic perfusion of porcine lungs resulted in reduced T cell infiltration post-allotransplantation compared to controls [137].

We know that inflammatory cytokines are released during kidney perfusion, although it is currently unclear what cytokines are detectable and whether these would be predictive of outcome. The use of a cytokine filter during 6 hours of normothermic perfusion of porcine kidneys reduced levels of interleukin-8 (neutrophil-attractant) and interleukin-6 (pro-inflammatory cytokine) when compared to control [138]. However, neither a difference in kidney function during normothermic perfusion could be noted nor were kidneys transplanted [138].

Ex situ organ perfusion offers an ideal platform to study and assess the extent of sterile inflammation. Models could make use of whole blood and reproduce a more complete inflammatory reaction. Studying sterile inflammation in the absence of circulating leukocytes and feedback loops from other organs, and tissues could also provide a valuable insight. As the inflammatory cascade takes changes over time and stretches from minutes to hours and day, prolonged perfusion could be valuable. In that light, it is worthwhile to mention that 24-hour perfusion of discarded kidneys has been described [55], and recently, discarded human livers have been sustained ex situ for one week on normothermic perfusion [139].

## 4. Conclusions

The need for adequate kidney viability and quality assessment tools is high, as acceptance or discard of a kidney remains a rather subjective decision. Dynamic kidney preservation offers an attractive way to study different aspects of an organ during preservation and to assess potential markers that portray information on organ viability and quality. This review has focused on the many different markers that have been proposed, some of which have been extensively investigated already. To date, however, none have reached the stage where they can reliably predict posttransplant outcome. This should not be surprising when considering the intricate interplay of the different kidney compartments, together with our lack of understanding on how ex situ perfusion might change this. Assessment of the entire kidney—the severity of injury, the remaining functional capacity, and the capacity to regenerate—by one biomarker seems highly unlikely. Much more reasonable would be to develop panels of biomarkers as we can learn from the quest for biomarkers of rejection [140].

With the dissemination of a variety of preservation techniques, international registries that collect a variety of perfusion settings and parameters would be of great value. Ideally, collaborative efforts to set up biobanks of perfusate, tissue, and urine are needed to allow discovery and derivation but also validation of any (set of) markers that are correlated with and predictive of outcomes. For example, the power of adding transcriptomic data to conventional risk models has been demonstrated to improve stratification of antibody rejection in kidney transplant recipients at high risk for graft loss [141].

The maturing organ perfusion technology and its dissemination in the clinical setting will lead to a deeper understanding of the behaviour of isolated organs during perfusion and how this behaviour might predict outcomes after transplantation. We will need to understand how the organ functions away from the organism that sustains it, so that we might offer it the ideal combination of nutrients and other drugs to improve organ preservation techniques, organ viability assessment, and ultimately organ repair and manipulation. Technological advances in the *-omics* field will play a major role in developing this crucial understanding.

## Figures and Tables

**Table 1:** Kidney quality assessment score for kidneys undergoing 1 hour of preimplantation normothermic perfusion [121].

	**Score**
**Macroscopic assessment**	
Grade I: excellent perfusion (global pink appearance)	1
Grade II: moderate perfusion (patchy appearance)	2
Grade III: poor perfusion (global mottled and purple/black appearance)	3
**Renal blood flow (ml/min/100 g)**	
Threshold ≥ 50	0
Threshold < 50	1
**Total urine output (ml)**	
Threshold ≥ 43	0
Threshold < 43	1
